# Classification and biomarker gene selection of pyroptosis-related gene expression in psoriasis using a random forest algorithm

**DOI:** 10.3389/fgene.2022.850108

**Published:** 2022-08-30

**Authors:** Jian-Kun Song, Ying Zhang, Xiao-Ya Fei, Yi-Ran Chen, Ying Luo, Jing-Si Jiang, Yi Ru, Yan-Wei Xiang, Bin Li, Yue Luo, Le Kuai

**Affiliations:** ^1^ Shanghai Skin Disease Hospital, School of Medicine, Tongji University, Shanghai, China; ^2^ Department of Dermatology, Yueyang Hospital of Integrated Traditional Chinese and Western Medicine, Shanghai University of Traditional Chinese Medicine, Shanghai, China; ^3^ Institute of Dermatology, Shanghai Academy of Traditional Chinese Medicine, Shanghai, China; ^4^ School of Rehabilitation Science, Shanghai University of Traditional Chinese Medicine, Shanghai, China

**Keywords:** pyroptosis, pyroptosis-related genes, psoriasis, random forest algorithm, machine learning

## Abstract

**Background:** Psoriasis is a chronic and immune-mediated skin disorder that currently has no cure. Pyroptosis has been proved to be involved in the pathogenesis and progression of psoriasis. However, the role pyroptosis plays in psoriasis remains elusive.

**Methods:** RNA-sequencing data of psoriasis patients were obtained from the Gene Expression Omnibus (GEO) database, and differentially expressed pyroptosis-related genes (PRGs) between psoriasis patients and normal individuals were obtained. A principal component analysis (PCA) was conducted to determine whether PRGs could be used to distinguish the samples. PRG and immune cell correlation was also investigated. Subsequently, a novel diagnostic model comprising PRGs for psoriasis was constructed using a random forest algorithm (ntree = 400). A receiver operating characteristic (ROC) analysis was used to evaluate the classification performance through both internal and external validation. Consensus clustering analysis was used to investigate whether there was a difference in biological functions within PRG-based subtypes. Finally, the expression of the kernel PRGs were validated *in vivo* by qRT-PCR*.*

**Results:** We identified a total of 39 PRGs, which could distinguish psoriasis samples from normal samples. The process of T cell CD4 memory activated and mast cells resting were correlated with PRGs. Ten PRGs, IL-1β, AIM2, CASP5, DHX9, CASP4, CYCS, CASP1, GZMB, CHMP2B, and CASP8, were subsequently screened using a random forest diagnostic model. ROC analysis revealed that our model has good diagnostic performance in both internal validation (area under the curve [AUC] = 0.930 [95% CI 0.877–0.984]) and external validation (mean AUC = 0.852). PRG subtypes indicated differences in metabolic processes and the MAPK signaling pathway. Finally, the qRT-PCR results demonstrated the apparent dysregulation of PRGs in psoriasis, especially AIM2 and GZMB.

**Conclusion:** Pyroptosis may play a crucial role in psoriasis and could provide new insights into the diagnosis and underlying mechanisms of psoriasis.

## 1 Introduction

Psoriasis is regarded as an immune-mediated, inflammatory skin disease (Demir Pektas et al., 2018), affecting more than 100 million people worldwide; estimates of its prevalence vary from 0.51 to 11.43% (Michalek et al., 2017). Comorbidities associated with psoriasis include cardiovascular disease (Tangtatco and Lara-Corrales, 2017), chronic obstructive pulmonary disease (Ungprasert et al., 2016), and metabolic syndrome (Armstrong et al., 2013), which can affect a patient’s quality of life and pose significant challenges for the medical field.

**FIGURE 1:**
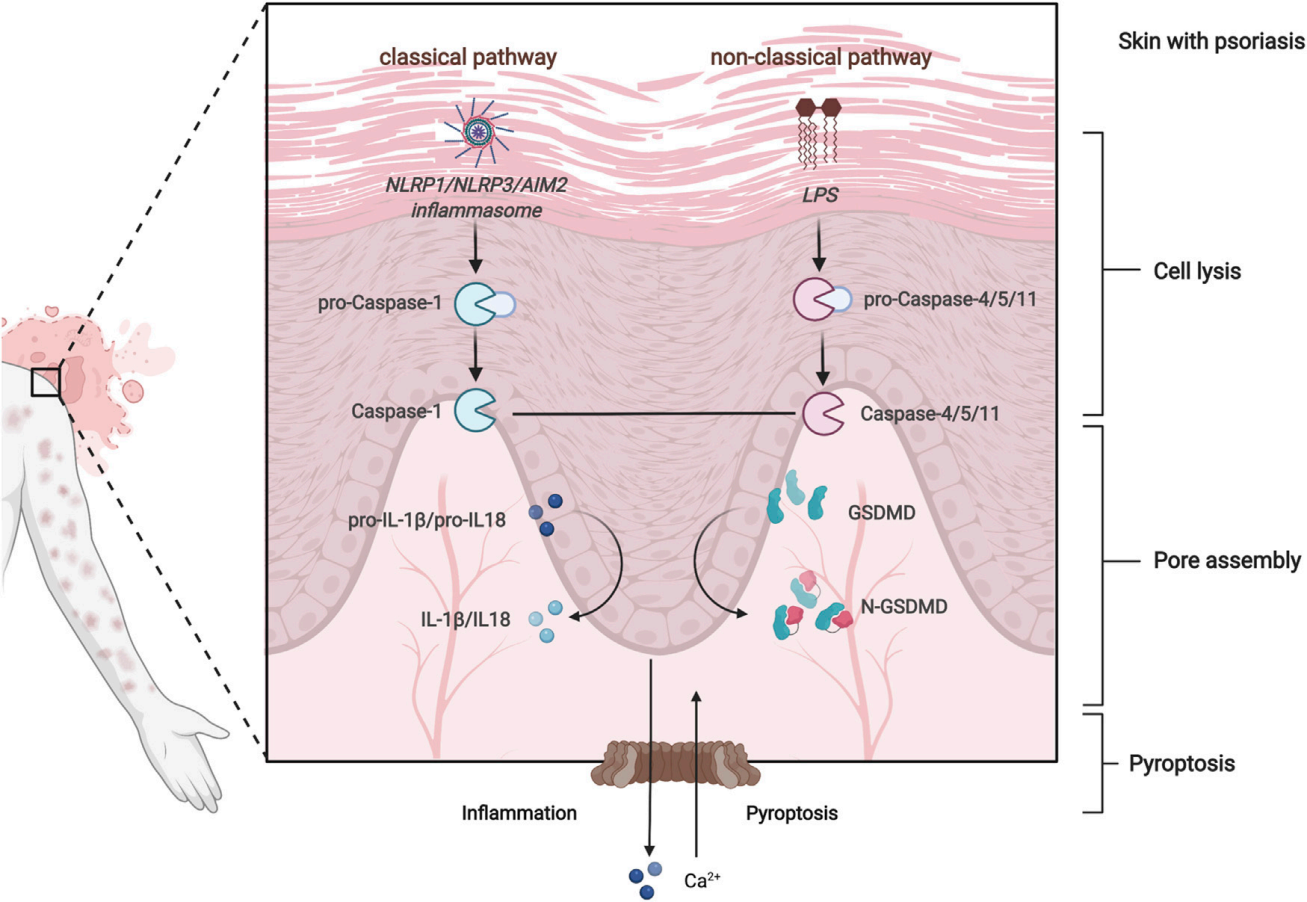
Overview of pyroptosis regulatory pathways.

Psoriasis is thought to progress as a result of abnormal keratinocyte cell death in the skin (Ramirez et al., 2012). The unique keratinization process of psoriasis is that late differentiation markers are abolished because of premature cell death (Iizuka et al., 2004), including pyroptosis, apoptosis, necrosis, necroptosis, and ferroptosis. Pyroptosis-related genes (PRGs) have been shown to have a strong association with aberrant keratinocyte keratinization *in vitro* (Lachner et al., 2017). Pyroptosis is a novel, inflammation-dependent type of programmed cell death (Fang et al., 2020), encompassing the classical caspase-1 pathway and the non-classical caspase-4/5/11 pathway (Song et al., 2021) (Figure 1). As studies continue, the non-classical pathway, caspase-11-mediated pyroptosis has been found to contribute to the pathogenesis of psoriasis (Kenealy et al., 2019). Multiple pieces of evidence indicated that PRGs are differentially expressed in psoriasis, such as GSDMD, caspase-1/2, IL-1 family genes (Lachner et al., 2017), NLRP1, NLRP3 (Deng et al., 2019), and AIM2 (Ciążyńska et al., 2021). These findings suggest that pyroptosis may serve as a potential prognostic or therapeutic target in psoriasis.

Due to the limitations of current investigational techniques, however, relevant gene expression studies have often been confined to one or two PRGs, despite psoriasis being a polygenically mediated disorder (Georgescu et al., 2019). A comprehensive understanding of PRG characteristics will be of great importance in elucidating the underlying mechanisms and predicting the response of psoriasis to immunotherapy. Hence, there is an urgent need to establish a predictive screening model to identify potential PRGs of psoriasis with a high predictive accuracy.

Various machine learning models have shown encouraging performance in biomarker prediction studies (Izadyyazdanabadi et al., 2018). The random forest model, one of the best conventional machine learning approaches (Jha et al., 2018) (Douville et al., 2021), is based on the principle of ensemble learning (Sessa et al., 2020), which has previously shown a high predictive accuracy (62–71%) in modeling (Tran et al., 2019) and provide variable importance estimates than classifiers. The random forest method has advantages even in uninformative predictor of random forest missing data mechanism (Moore et al., 2015). Despite the potential usefulness of random forest, its application in psoriasis PRG regulators have not been reported.

Here, we comprehensively evaluated the regulation of PRGs. First, the differential expressions of PRGs between psoriasis and normal samples was analyzed. Second, a machine learning model of psoriasis was established using a random forest algorithm to innovatively identify potential therapeutic approaches. Third, the relationship between pyroptosis and the immune system was investigated. Subsequently, we validated the mRNA expression of PRGs *in vivo*. Therefore, this research provides potential targets for the diagnostic and treatment of patients with psoriasis.

## 2 Methods

### 2.1 Psoriasis patients’ dataset

#### 2.1.1 mRNA expression profile of psoriasis patients

The mRNA expression profiles of psoriasis patients and control were downloaded from the Gene Expression Omnibus (GEO) database (Barrett et al., 2013) (http://www.ncbi.nlm.nih.gov/geo/). The data acquisition criteria were as follows: all participants of the included studies were human; samples were not based on cell lines; sample type should be skin tissue; patients should be diagnosed with psoriasis; datasets should be complete data for analysis; ethical approval was obtained; and the control group had neither systemic nor autoimmune diseases and no relevant family history. GSE114286 was obtained as the training cohort, and the other three datasets (GSE14905, GSE109248, and GSE117239) were obtained for external validation analyses, the basic information for which is shown in Table 1.

**TABLE 1:** Basic information of included datasets.

GEO ID	Sample numbers	PMID	Number of samples		Age		Gender (M/F)		Tissue type	Microarray platform	Array type	RNA-seq method	Number of DEGs
			Psoriasis	Control	Psoriasis	Control	Psoriasis	Control					
GSE114286	27	30341238	18	9	43.83 ± 16.29	37.33 ± 13.73	12/6	6/3	skin tissue	GPL17303	—	High-throughput sequencing	2139
GSE14905	82	18648529	61	21	—	—	—	—	skin tissue	GPL570	Affymetrix Human Genome U133 Plus 2.0 Array	—	2873
GSE109248	56	29889098	17	14	—	—	—	—	skin tissue	GPL10558	Illumina HumanHT-12 V4.0 expression beadchip	—	1860
GSE117239	324	30703387	240	84	—	—	—	—	skin tissue	GPL570	Affymetrix Human Genome U133 Plus 2.0 Array	—	979

#### 2.1.2 Dataset characteristics

The R package limma was adopted to identify the differentially expressed genes (DEGs) between psoriasis and normal samples. To correct for false-positive results, Benjamini and Hochberg’s (1995) false discovery rate (FDR) method was used by default. A fold change of >1.5, a *p*-value <0.05, and an FDR <0.05 were set as the cutoffs to screen DEGs (Yu et al., 2012).

### 2.2 Characteristics of PRGs

#### 2.2.1 PRGs obtained

Two pyroptosis gene sets were acquired (REACTOME_PYROPTOSIS and GOBP_PYROPTOSIS), and a total of 39 PRGs were obtained from the Molecular Signatures Database (MSigDB) (Liberzon et al., 2015) (https://www.gsea-msigdb.org/gsea/msigdb/index.jsp).

#### 2.2.2 PRG internal correlation analysis and protein–protein interaction (PPI) analysis

For correlation analysis among PRGs, the Spearman correlation function of the R package was used. The range of the correlation coefficient of a pair (r) was from 1 to -1; r values close to 1 or -1 indicate a strong positive or negative correlation between genes, respectively.

A protein–protein interaction (PPI) network analysis was performed to identify potential interactions among PRGs. The STRING database (Szklarczyk et al., 2017) (https://string-db.org/) was used to obtain interaction pairs for proteins related to pyroptosis with the highest confidence (interaction minimum >0.9). Cytoscape software (Shannon et al., 2003) (version 3.9.0) was then used to display the PPI network.

### 2.3 Identification of differentially expressed PRGs

#### 2.3.1 DE mRNA levels of PRGs

Pyroptosis-related genes were extracted, and we screened the PRGs using the R package limma, with *p* < 0.05, |Fold change|>1.5 as the conditions. The heatmap was created using the R packages pheatmap and ggplot2 to show differentially expressed PRGs in patients with psoriasis compared with normal control samples. We also examined the effectiveness of PRGs in other skin diseases.

#### 2.3.2 The ability of differentially expressed (DE) PRGs to discriminate samples by principal component analysis (PCA)

To determine whether PRGs were suitable for distinguishing psoriasis from normal tissue samples, PCA was used to determine the genotyping consistency using the R package mixomics.

### 2.4 PRGs and immune cell correlation representation

Pearson correlation were performed for PRGs and immune cell abundance using the cor function in R, and then the pheatmap function was used to draw a heatmap. If the grid on the right image is marked with *, it means that the absolute value of the correlation between the pyroptosis gene and immune cells is >0.5.

### 2.5 Diagnostic model using a random forest algorithm

#### 2.5.1 Establishing the random forest model

We used a machine learning diagnostic model, the random forest, to screen candidate psoriasis-specific genes from PRGs using the R package randomForest (Liaw and Wiener, 2002). Random forest is an ensemble method for the training and prediction of samples based on multiple classification trees. The best split among all variables was used for each node, and the best among a random subset of predictors was used for the random forest. The parameter optimization is initially performed using randomly generated parameter sets. As computation time increases with increasing ntree, we used ntree = 400 in our modeling. The training set contained three quarters of the samples in each repetition. The procedure was repeated 100 times by random sampling.

#### 2.5.2 Internal validation

Conventionally, the internal validation set should include one quarter of the samples in each repetition. Ten-fold cross-validation was performed as an internal validation method to confirm the predictive performance of our diagnostic model for psoriasis based on PRGs. The receiver operating characteristic (ROC) curves were generated, and the area under the curves (AUC) were derived as the internal cross-validation, using the pROC package in R to evaluate the discriminative ability.

#### 2.5.3 External validation

The universality and reliability of the random forest diagnostic model were independently validated against an external validation cohort, including the GSE14905, GSE109248, and GSE117239 datasets, whose characteristics are shown in Table 1. The ROC analysis procedure was performed.

### 2.6 Consensus clustering analysis of PRGs

We performed consensus clustering to investigate whether there was a difference within PRG-based subtypes, such as psoriasis-related functions and pathways. Consensus clustering is an unsupervised clustering algorithm used to identify subgroup members and verify subgroups based on resampling.

#### 2.6.1 Identification of pyroptosis-related subtypes

The classification of patients into subtypes based on PRGs was calculated using the R packages consensusclusterplus and pheatmap, which were employed for consensus unsupervised clustering analysis. The box plots were created using the R software package ggplot2 (Yi et al., 2020). The criteria included cumulative distribution function (CDF) curve increasing smoothly. The k value was adopted to describe the number of clusters, from 2 to 8 and finally set k = 3 as the optimal subtype number because of the highest delta area score.

#### 2.6.2 DEGs based on PRG subtypes

The consensus clustering classified psoriasis into three distinct subtypes based on PRGs. We next identified DEGs among the different clusters using the limma package. Considering the insufficient sample size in cluster 3 (sample duplicates <3), we focused on the first two clusters.

#### 2.6.3 Biological functions based on PRG subtypes

To explore the differentiation in biological characteristics and potential pathways between PRG-based subtypes, the Gene Ontology (GO) and Kyoto Encyclopedia of Genes and Genomes (KEGG) pathways among clusters were investigated using the R package clusterProfiler (Kursa, 2014), with an FDR cutoff of <0.01.

### 2.7 Experimental verification

#### 2.7.1 Animal preparation

Twelve male C57BL/6 mice, aged 6–7 weeks, weighing 22–25 g, provided by Shanghai Medical Experimental Animal Center (permission no. SCXK(Hu)2018-0003, Shanghai, China) were used as experimental animals. They were housed in a specific pathogen free (SPF) facility, at a temperature of 21–25°C, 16–8 h light–dark cycle, free water, and standard diet provided by Shanghai Pu Lu Tong Biological Technology Co., Ltd. The Yueyang Hospital of Integrated Traditional Chinese and Western Medicine of Shanghai University of Traditional Chinese Medicine Ethics Committee approved the animal experiment (No. YYLAC-2020-078-3) (Supplementary File S1).

#### 2.7.2 Imiquimod (IMQ)-induced psoriasis-like mice model establishment and intervention

After shaving the hair of mice in a 2 × 2 cm^2^ square area, the animals were randomly separated into two groups:

1) Control group (NC), received a topical dose (62.5 mg) of petroleum jelly.
2) Psoriasiform modeling group (IMQ), received a topical dose (62.5 mg) of IMQ cream (5%) for 6 h prior to intragastric administration of saline (1.8 g/kg).

All treatments were applied once per day for 12 consecutive days from the date of application of IMQ (day 0). Mice were fasted and allowed to drink water for 12 h prior to sample collection. The animals were euthanized by CO_2_ inhalation (day 12), and back and ear tissues were collected for further experiments.

#### 2.7.3 qRT-PCR

To detect the mRNA expression level of PRGs selected by the random forest algorithm, qRT-PCR was used. Skin tissue homogenates from psoriasis-like mice were taken and applied Trizol reagent (Beyotime, China) protocol for total RNA extraction, whose concentration and purity were determined using an ultraviolet spectrophotometer and assessed via agarose gel electrophoresis. The relative quantitative method (2^−ΔΔCT^) was applied. Reverse transcriptase was used to prepare cDNA. The results were normalized to GAPDH. Primers were designed and synthesized (Table 2).

**TABLE 2:** Primers used for qRT-PCR.

	Primer sequence
IL1B	F:5′TGGACCTTCCAGGATGAGGACA3′
	R:5′GTTCATCTCGGAGCCTGTAGTG3′
AIM2	F: 5′AGG​CTG​CTA​CAG​AAG​TCT​GTC​C3′
	R: 5′TCA​GCA​CCG​TGA​CAA​CAA​GTG​G3′
DHX9	F: 5′AGG​GTC​CAG​TGG​AGA​CTA​CC3′
	R: 5′CCA​CCT​CCA​TAA​CCC​CTT​CG3′
CASP4	F:5′GTGGTGAAAGAGGAGCTTACAGC3′
	R:5′GCACCAGGAATGTGCTGTCTGA3′
CYCS	F:5′CACCGACACCGGTACATAGG3′
	R: 5′TAA​TTC​GTT​CCG​GGC​TGG​TC3′
CASP1	F:5′GAAACGCCATGGCTGACAAG3′
	R: 5′GAT​CAC​ATA​GGT​CCC​GTG​CC3′
CASP8	F:5′ATGGCTACGGTGAAGAACTGCG3′
	R: 5′TAG​TTC​ACG​CCA​GTC​AGG​ATG​C3′
CHMP2B	F:5′CGAGCAGCCTTAGAGAAACAGG3′
	R: 5′GTT​TCC​GTA​GGT​GGA​CAA​GCT​G3′
GZMB	F:5′CAGGAGAAGACCCAGCAAGTCA3′
	R: 5′CTC​ACA​GCT​CTA​GTC​CTC​TTG​G3′

#### 2.7.4 Statistical methods

All experimental data were analyzed using the statistical software SPSS 24.0 (IBM Corp., Armonk International Business Machines, New York, United States). Data are presented as mean ± standard deviation (SD). Comparisons between two groups were conducted using one-way ANOVA. Significant differences among two groups were indicated by **p < 0.01, *p < 0.05, or ***p < 0.001, two-sided.

## 3 Results

**FIGURE 2:**
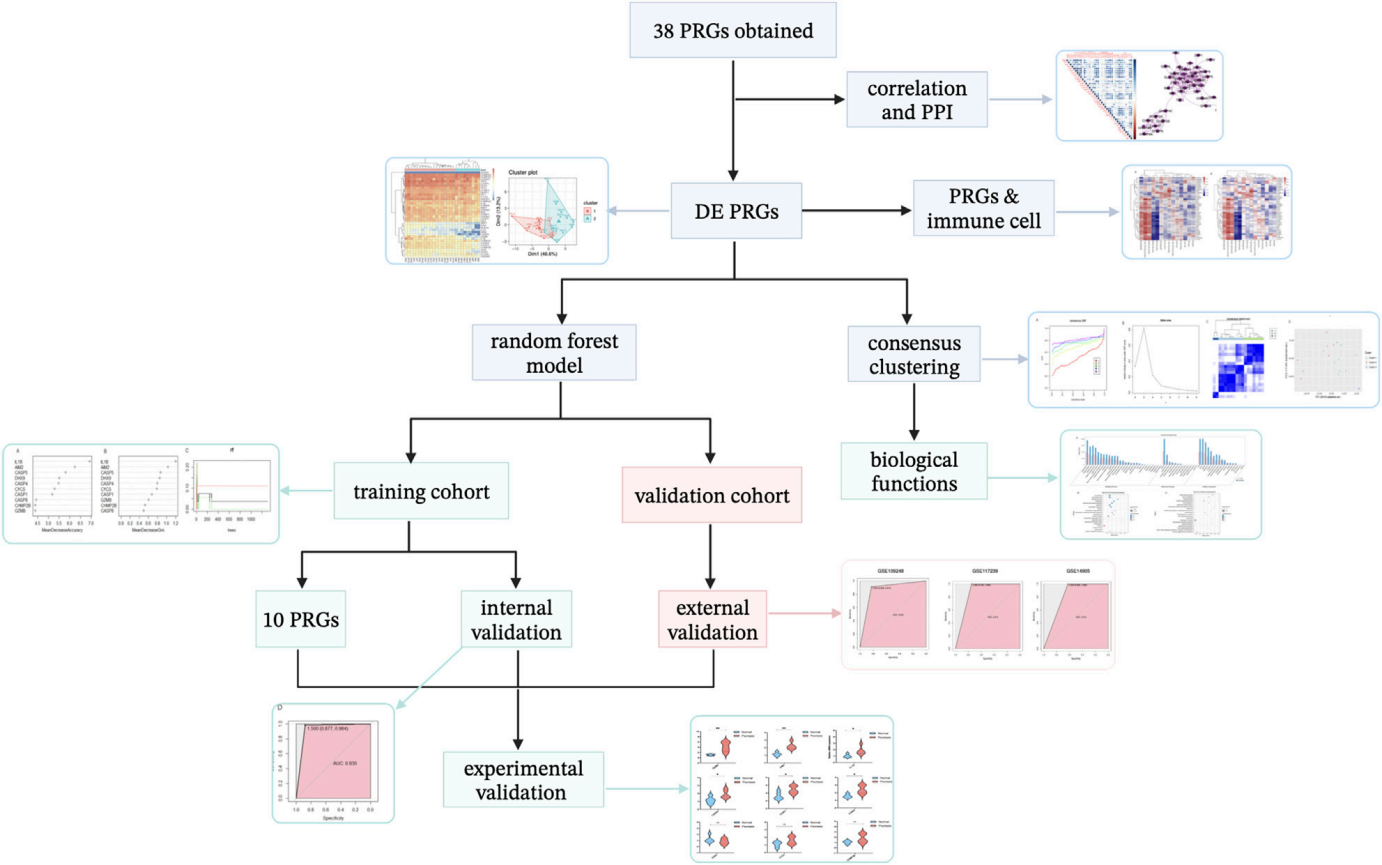
Article framework and workflow. PRGs, pyroptosis-related genes. PPI, protein–protein interaction. DE, differentially expressed.

A flow diagram representing this work is shown in Figure 2. First, the characteristic information and gene expression profiles of psoriasis cases and healthy controls in the GSE114286, GSE14905, GSE109248, and GSE117239 datasets were obtained. Using the GSE114286 dataset, we identified several differentially expressed PRGs, and PCA analysis indicated that the PRGs could distinguish psoriasis from normal tissue samples. Then, a correlation analysis between PRGs and immune cell were conducted as well.

Subsequently, we developed a novel diagnostic model for psoriasis based on PRGs using a machine learning random forest method, which screened 10 potential PRG biomarkers. GSE14905, GSE109248, and GSE117239 were used for external validation.

To discover whether there was a difference between subtypes of PRGs, we used consensus clustering analysis to divide the samples into three subtypes. The particular biological characteristics among clusters were investigated.

Finally, experimental validation was performed using IMQ-induced psoriasis-like mice and qRT-PCR analysis.

### 3.1 The transcriptional regulator landscape in psoriasis patients

The training cohort, the GSE98793 dataset, included 2,139 DEGs comparing psoriasis patients with healthy individuals and 39 PRGs were presented respectively (Supplementary Figure S1A). The external validation cohort (GSE14905, GSE109248, and GSE117239) included 1,959 DEGs (Supplementary Figure S1B), the basic information of which is shown in Table 1.

### 3.2 PRG characteristics

**FIGURE 3:**
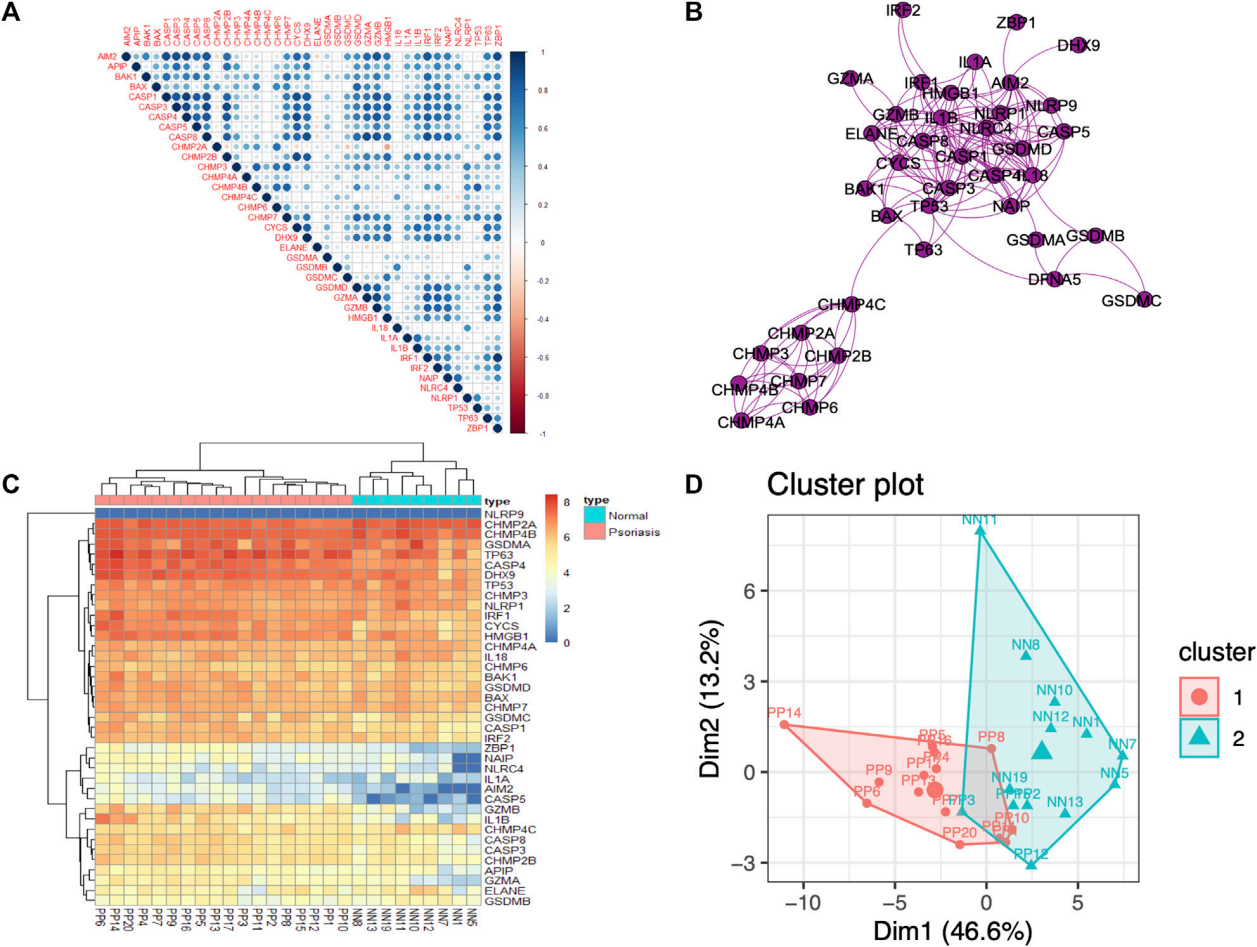
PRGs correlation and interaction analysis, and DE PRGs identification between psoriasis and normal samples **(A)**. Spearman correlation analysis of the 39 differentially expressed PRGs; blue represents a positive correlation, red represents a negative correlation, and the darker the color, the stronger the correlation. **(B)**. Protein–protein interaction (PPI) analysis of the 39 differentially expressed PRGs. **(C)**. Heatmap of the 39 differentially expressed PRGs in psoriasis and normal samples. **(D)**. Principal component analysis (PCA) of PRGs.

A total of 39 PRGs were obtained from MSigDB, and the annotation analysis is shown in Supplementary Figure S2. We attempted to demonstrate the PRGs correlation, and the results indicated that the PRGs have positive connection, especially AIM2, ACSP1, CASP8, and GZMB (Figure 3A). The interaction relationships of these PRGs regulators were exhibited as a PPI network, which reminded us that the PRGs have strong protein-protein interaction, especially in CHMPs family or CASPs family (Figure 3B). Among all nodes, CASP1 and IL1B had the highest degree of protein interactions in the PPI network. These results indicated that expression imbalances of PRGs played critical roles in psoriasis.

### 3.3 Identification of DE PRGs

#### 3.3.1 PRGs are differentially expressed in psoriasis

To investigate the contribution of PRGs to the pathogenesis of psoriasis, the mRNA expression of PRGs was compared between psoriasis and normal samples (Figure 3C). Furthermore, the results of differences on PRGs in other skin disorders, including psoriatic arthritis (GSE61281), atopic dermatitis (GSE124700 and GSE60709), and acne (GSE53795 and GSE6475), indicated that PRGs could be biomarkers of psoriasis (Supplementary Table S1).

#### 3.3.2 DE PRGs have the ability to discriminate psoriasis samples

The PCA results were used to determine distinctiveness-based confidence measures of PRGs (Dim1 = 46.6%, Dim2 = 13.2%), which indicated that PRGs have the ability to clearly distinguish psoriasis from normal samples (Figure 3D).

### 3.4 PRGs and immune cell correlation analysis

**FIGURE 4:**
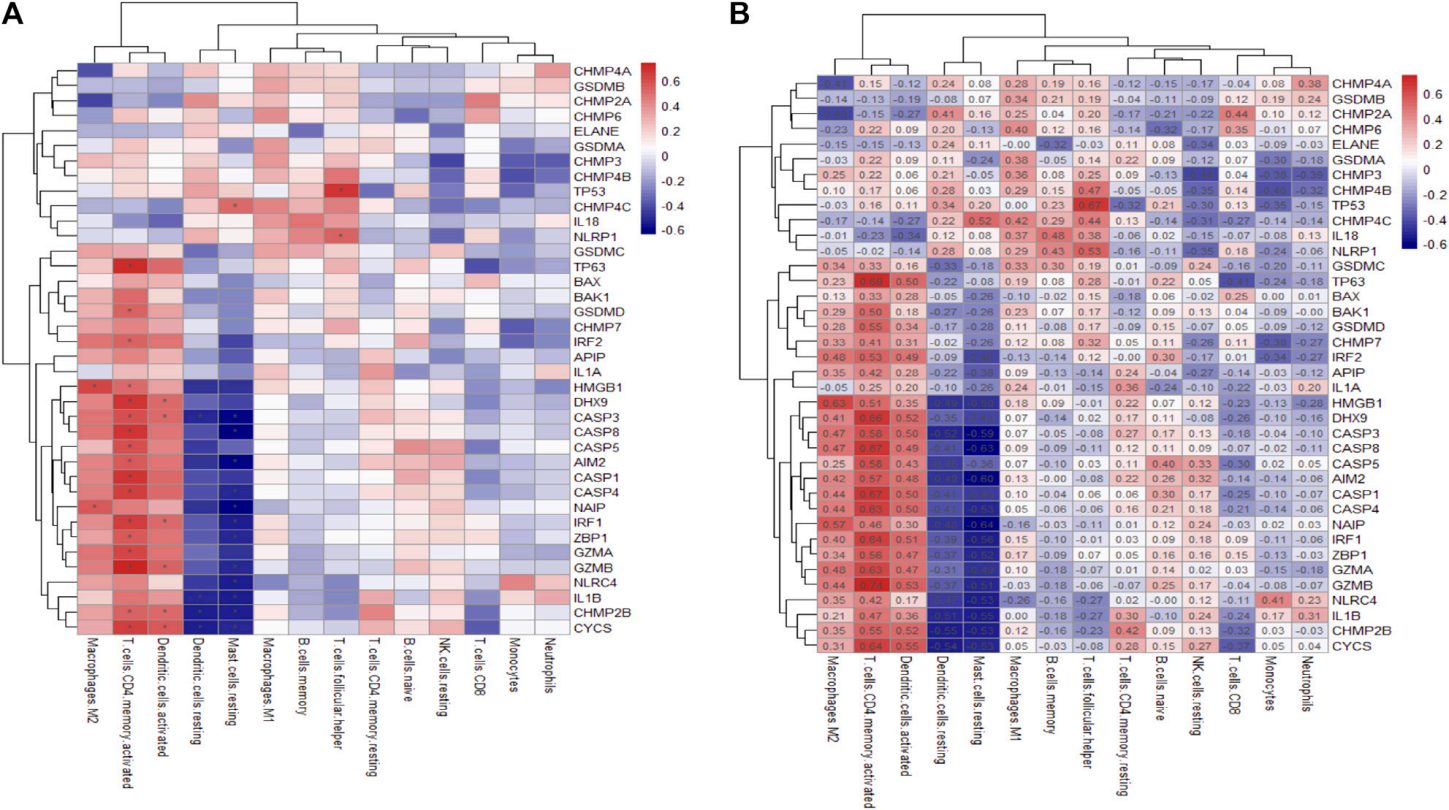
Heatmap of the differentially expressed PRGs in various immune cells. **(A)**. PRG correlation map with immune cells, where the horizontal axis represents PRGs and the vertical axis represents immune cells. **(B)** The correlation map marked with absolute value. Asterisks represent levels of significance *p < 0.05.

Emerging evidence has indicated there is crosstalk between pyroptosis and immune responses (Orning et al., 2019). In Figure 4, T cells CD4 memory activated show the strongest positive correlation with TP63 (r = 0.69), while mast cells resting and NAIP are the most negatively correlated pair (r = -0.64). The CIBERSORT and immune cell abundance between psoriasis and normal is shown in Supplementary Figure S3.

### 3.5 Random forest model mediated by PRGs

#### 3.5.1 Ten feature DE PRGs were screened out random forest model

**FIGURE 5:**
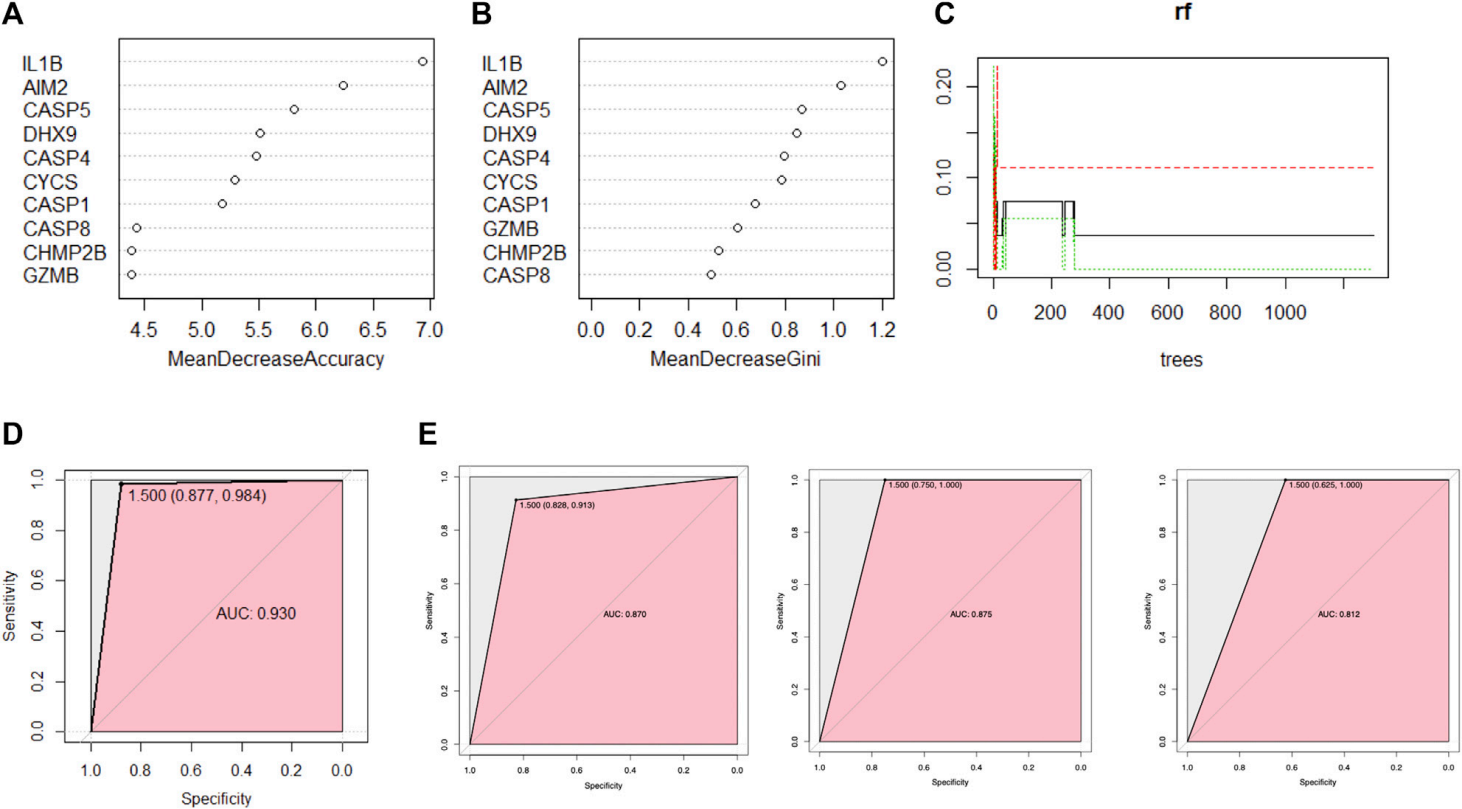
A random forest algorithm was used for ranking the importance of prognostic PRGs. **(A,B)** The importance of PRGs using the scores returned by the random forest model. **(C)** Parameter optimization was initially performed using randomly generated parameter sets, ntree = 400 was selected in the modeling, and stability was achieved when 100 random samples were taken, three quarters of the dataset was grouped as the training set and the remaining one quarter as the validation set in each repetition. **(D)** The receiver operating characteristic (ROC) curve (area under the curve (AUC) = 0.93) was generated by cross-internal validation. **(E)** ROC curve on independent external validation, from left to right are GSE117239, GSE109248, and GSE14905, respectively.

The random forest algorithm was used to rank the importance of prognostic PRGs. Applying the selection criteria previously used, we shortlisted 10 PRGs by mean decrease accuracy and mean decrease gini (Figures 5A–C). Interleukin 1 beta (IL-1β) was the most prominent pyroptosis eigengene of psoriasis, followed by absent in melanoma 2 (AIM2) and caspase 5 (CASP5). DExH-Box helicase 9 (DHX9) and caspase 4 (CASP4) contain slightly more predominance than cytochrome C, somatic (CYCS), and caspase 1 (CASP1).

#### 3.5.2 Internal validation

The model demonstrated good discrimination by performing cross-validation in an ROC curve in internal validation [AUC = 0.930 (0.877, 0.984)] (Figure 5D), which indicated the excellent performance of the current random forest model.

### 3.5.3 External validation

The ROC curve analysis was subsequently used for independent external validation, with the value of the AUC representing the consistency of the model. The results showed that the AUC was 0.870 (95% CI 0.828–0.913) for GSE109248, 0.875 (95% CI 0.750–1.000) for GSE117239, and 0.812 (95% CI 0.625–1.000) for GSE14905 (Figure 5E), which indicated the model had a good predictive performance. In summary, this random forest model showed reliable predictive power in the external discrimination test (mean AUC = 0.852).

### 3.6 Consensus clustering analysis of PRGs

#### 3.6.1 Three pyroptosis subtypes of psoriasis obtained

**FIGURE 6:**
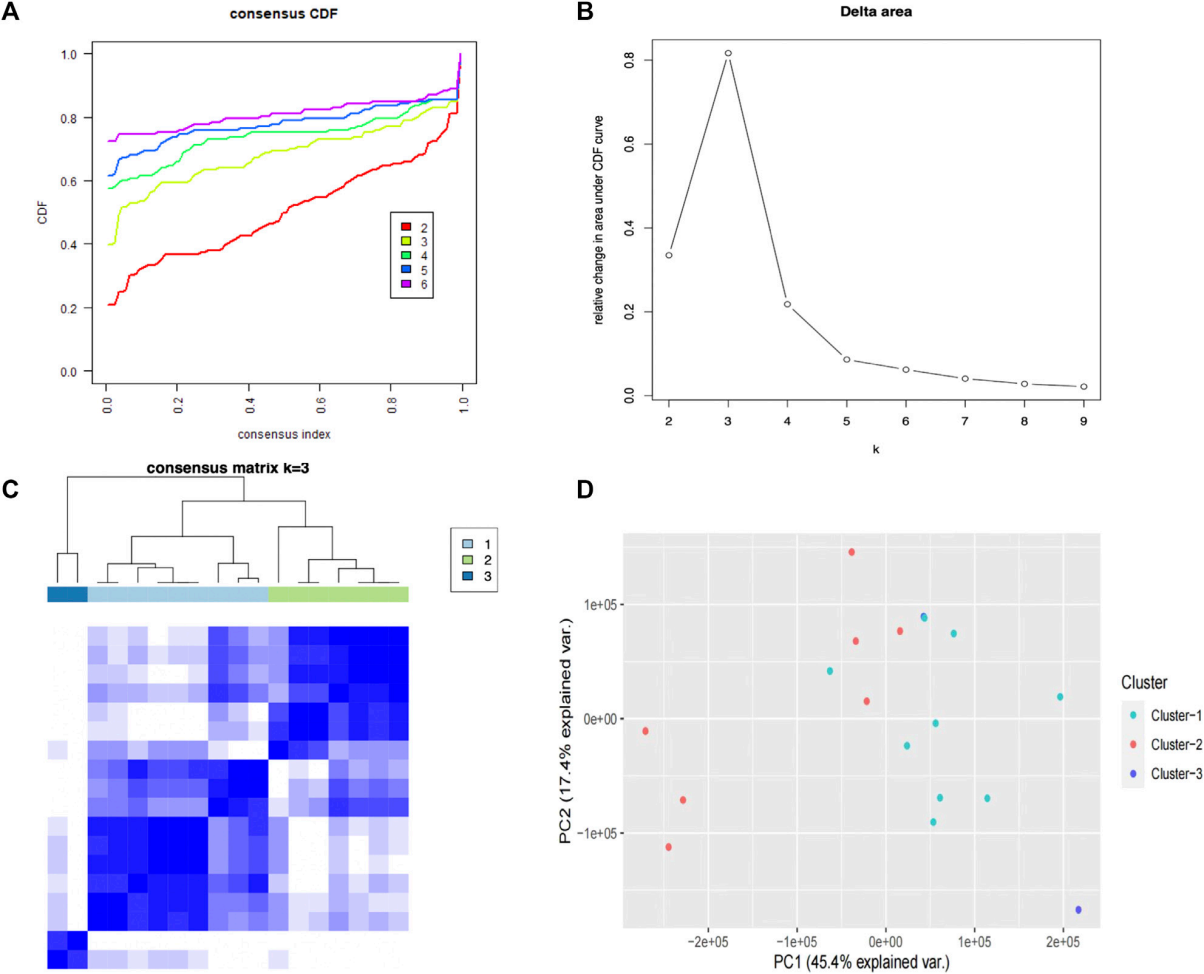
Consensus clustering analysis of PRGs. **(A)** The cumulative distribution function (CDF) curve for each category number k compared with k -1.**(B)** Delta area curve of consensus clustering, indicating the relative change in area under the CDF. The horizontal axis represents the category number k, and the vertical axis represents the relative change in area under the CDF curve. **(C)** A heatmap showing the consensus clustering solution (k = 3) for 39 PRGs in three clusters. **(D)** The PCA results show these three clusters were significantly differentiated, especially clusters 1 and 2.

To investigate the association between the PRGs and psoriasis subtypes, we used a consensus clustering algorithm in the training cohort. When variable (k) = 3, the psoriasis patients could be well separated into three subtypes of 39 PRGs (Figures 6A–C) with the highest intragroup correlations and the lowest intergroup correlations. Cluster 1 included nine patients and cluster 2 included seven patients, leaving two psoriasis patients in cluster 3. The PCA results showed these three clusters were significantly different from each other, especially cluster 1 and cluster 2 (Figure 6D).

#### 3.6.2 Differential genes and biological functions based on PRGs subtype

**FIGURE 7:**
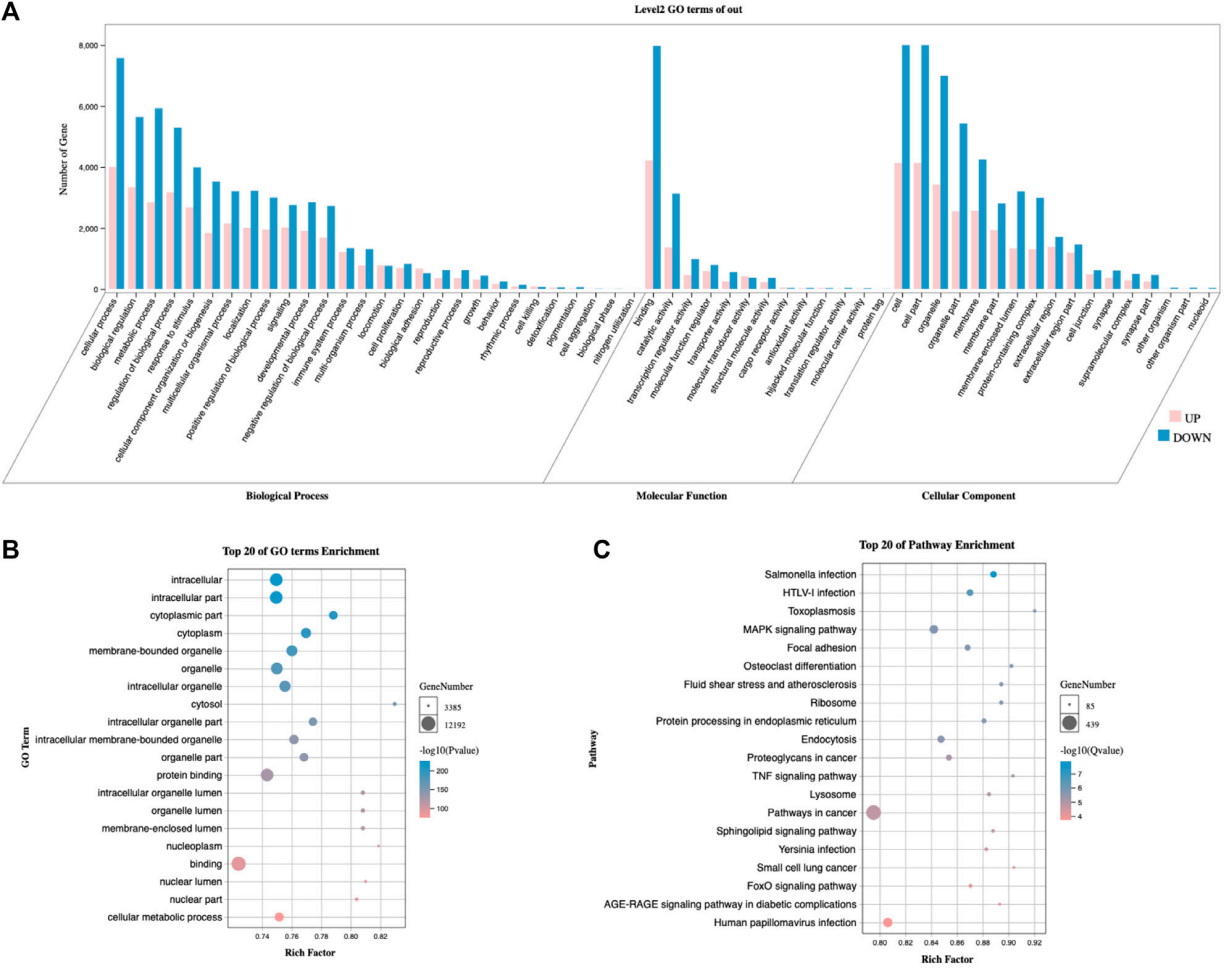
Comparison of biological functions analysis between clusters 1 and 2. **(A)** The secondary classification of GO, including biological processes, molecular functions, and cellular components. **(B)** Bubble map of the GO analysis, ranked by *p*-value. **(C)** Bubble map of KEGG signaling pathways, ranked by *Q*-value. GO, Gene Ontology. KEGG, Kyoto Encyclopedia of Genes and Genomes.

To investigate the differences in biological functions between cluster 1 and cluster 2, we initially calculated the differentially expressed mRNAs (Supplementary Table S2). Next, we performed the GO and KEGG enrichment analyses, which revealed the enrichment of metabolic processes (Figures 7A,B), the MAPK signaling pathway, FoxO signaling, and the RAGE signaling pathway (Figure 7D).

### 3.7 qRT-PCR-verified expression of PRGs in IMQ-induced psoriasis-like mice

To further demonstrate the results of the random forest model, we validated the nine target mRNAs in psoriatic mice, except for CASP5, which is not present in mice. In contrast with the normal group, significant changes were observed by qRT-PCR in the expression of several PRGs in the psoriasis group.

**FIGURE 8:**
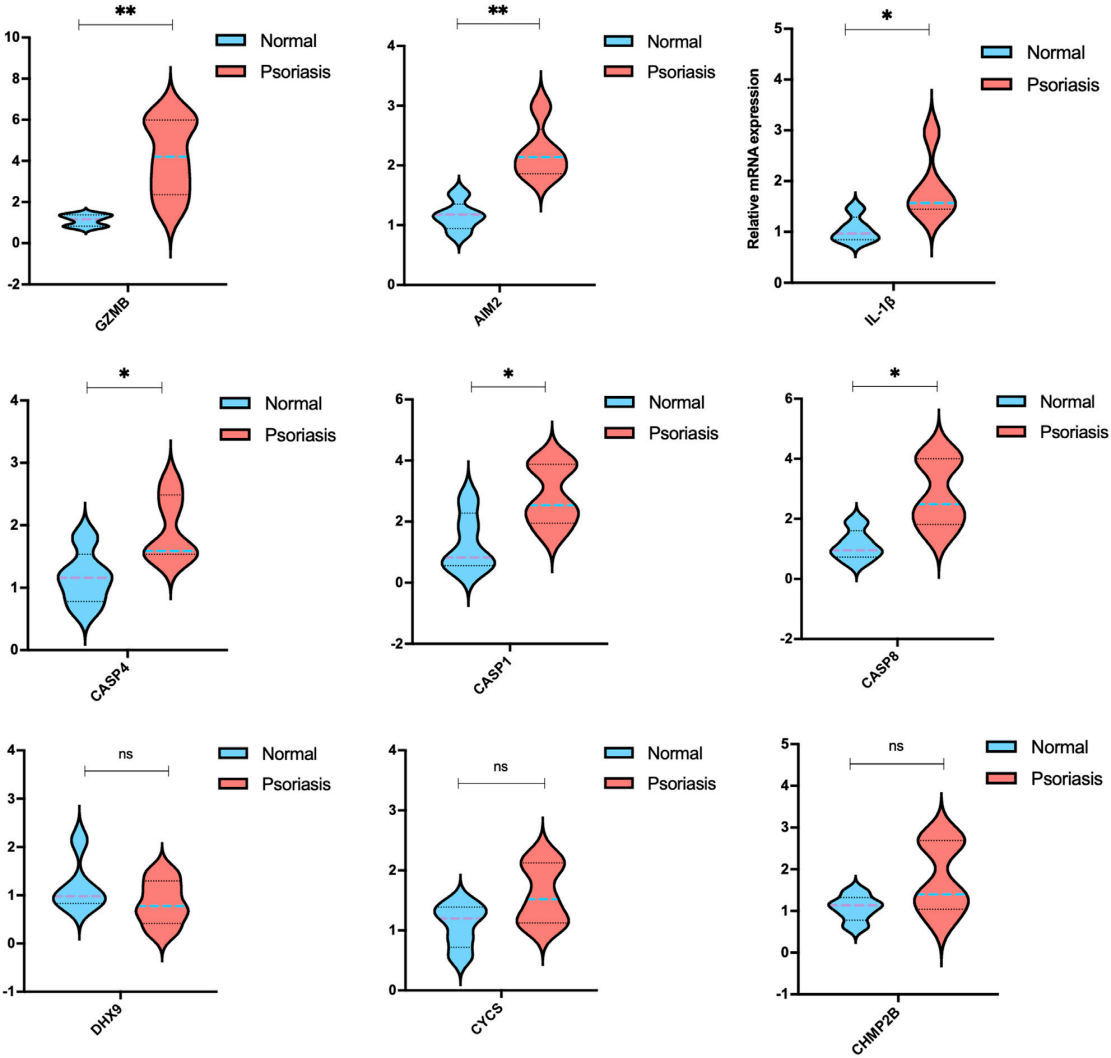
mRNA levels of the candidate PRGs of imiquimod (IMQ)-induced psoriasis-like mice compared with the control group (*n* = 5). The data are expressed as means ± SD. Four skin lesions in each group were included in the analysis. *p < 0.05, **p < 0.01, compared with the control group.

The most obvious significance of PRGs expression was observed in Granzyme B (GZMB) (Figure 8), which was dramatically elevated in inflammatory skin disorders; it is known for its pro-apoptotic function, as well as epithelial barrier disruption (Turner et al., 2019). Psoriasis has a high levels of AIM2, the activation of which initiates the assembly of the inflammasome, triggering the maturation and secretion of the cytokine IL-1β (Lugrin and Martinon, 2018, 2). Here, we revealed the high expression of the cysteinyl aspartate protease (caspase, or CASP) gene family, including CASP1, CASP4, and CASP8, which play significant roles in programmed cell death, inflammation, and immunity (Hong et al., 2020). However, no statistical significance was shown on DHX9, CYCS, and CHMP2B, which should be accomplished in future research using large scale clinical samples.

## 4 Discussion

Many studies have reported the indispensable role pyroptosis plays in psoriasis (Wang et al., 2020). Whereas most research focused on one or two PRGs *in vivo* or *vitro*, for instance, the pathogenesis of psoriasis is closely related to caspase-1, IL-1β, IL-18, or GSDMD. The contribution degree of PRGs in psoriasis are still elucidated.

Here, we identified several differentially expressed PRGs, and PCA indicated that these PRGs could distinguish psoriasis from normal tissue samples. Subsequently, we developed a novel diagnostic model for psoriasis based on PRGs, using the random forest machine learning method, which involved screening 10 potential PRG biomarkers, followed by internal and external validation. We also looked for correlations between immune cells and PRGs. To discover whether there was a difference between subtypes of PRGs, consensus clustering analysis was used to divide the samples into three subtypes. The particular biological characteristics among clusters were observed. Finally, experimental validation was performed using IMQ-induced psoriasis-like mice and qRT-PCR analysis.

Random forest, a machine learning algorithm, was the main predictive model used in our study to reveal contribution degree of PRGs in psoriasis. It is an ensemble machine learning algorithm based on decision trees, with strong anti-noise ability and good robustness, and can be used for feature selection based on features with a high prediction accuracy; it is widely used for a variety of classification and regression problems (Moore et al., 2015). We identified 10 regulators based on feature values from the random forest algorithm. Subsequent experimental validation provided further evidence that mRNA expression of AIM2, CASP4, CASP1, CASP8, and GZMB was significantly upregulated in IMQ-induced mice.

Pyroptosis is regulated by the classical caspase-1 pathway and the non-classical caspase-4/5/11 pathway (Xiang and Yang, 2020). Upon stimulation by pathogenic microbes, intracellular pattern recognition receptors (PRRs), such as AIM2, assemble into caspase-1-dependent inflammasomes via apoptosis-related specific protein pattern recognition receptors. Next, the activated caspase-1 cleaves the inflammatory cytokine IL-1β and IL-18 precursor, making it an active cytokine, which is the classical pathway of pyroptosis (Chen et al., 2018). Pyroptosis is characterized by the cellular production of IL-1β (Wagner et al., 2017). Also, the tendency of the numerical results to meet an agreement with our experiments. Our model revealed consistencies with clinical observations that the augmented protein levels of IL-1β in skin tissue are associated with psoriatic lesional area and severity (Cai et al., 2019) (Su et al., 2018, 3) (Yu et al., 2021) (Ciążyńska et al., 2021, 2). Enhanced expression of IL-1β mRNA has been observed *in vivo*, in IMQ-induced mice (Shou et al., 2021) (Deng et al., 2019) (Tang et al., 2021). This tendency was in accordance with the result of one previous cell experiment (Kim et al., 2021). Aberrant or excessive activation of the classical caspase-1 path is associated with many autoinflammatory, autoimmune, and metabolic diseases (Shi et al., 2015). One study revealed that inflammasome sensors, NLRP3, NLRP1, CASP1, and AIM2, enhanced expression in psoriasis patients (Verma et al., 2021). An *in vivo* study demonstrated significantly augmenting protein expression of AIM2 in IMQ-induced psoriasis-like mouse model (Chung et al., 2020).

The non-classical pathway of pyroptosis mediated by caspase-4 and caspase-5 was identified as kernel regulators in psoriasis by a random forest algorithm in the current study. Consistent with our findings, the non-classical inflammasome is a complex of bacterial lipopolysaccharide together with caspase-4, caspase-5, and caspase-11 (Liu and Lieberman, 2017), which has been proved to directly activated GSDMD-mediates pyroptosis process. After stimulation of cells with lipopolysaccharide from gram-negative bacteria, caspase-4/5/11 directly binds to lipid A of lipopolysaccharide or activates caspase-1 via the NLRP3 inflammasome pathway, which cleaves GSDMD to become GSDMD N-terminal and GSDMD C-terminal, which in turn causes cell membrane rupture and lysis as well as the release of pyroptosis-related factors such as IL-18 and IL-1β (Liu and Lieberman, 2017). Notably, the augmented levels of CASP8 and GAMB were observed in the psoriasis-like dermatitis model, although their roles in the pathogenesis of psoriasis have not yet been investigated.

Accumulating evidence suggests that pyroptosis-related regulators play a crucial role in psoriasis. Intervention in the pyroptosis-related pathway could be a potential target for the treatment of psoriasis. Our study had some limitations. First, due to the lack of information about clinical outcomes, we were unable to directly evaluate the prognostic value of the current model, therefore, further studies are needed. Second, achieving practical strategies for translating psoriasis risk-associated genetic variants into functional annotations and clinical applications remains challenging.

## 5 Conclusion

In summary, we used a machine learning model and experimental verification to investigate the role of PRGs in the pathogenesis of psoriasis. Through the experimental verification of 10 PRGs obtained by machine learning, it was found that the mRNA expression of IL-1β, AIM2, CASP4, CASP1, CASP8, GZMB, especially IL-1β, CASP1, CASP8, and GZMB, was ameliorated to alleviate the skin lesions IMQ-induced psoriasis-like mice, which provided potential therapeutic targets.

## Data Availability

The datasets presented in this study can be found in online repositories. Further inquiries can be directed to the corresponding authors.

## Supplementary material

SUPPLEMENTARY FILE S1 The laboratory animal ethics review approval of Yueyang Hospital of Integrated Traditional Chinese and Western Medicine of Shanghai University of Traditional Chinese Medicine Ethics Committee.

SUPPLEMENTARY TABLE S1 PRG mRNA expression level of other skin disease-related datasets: atopic dermatitis, psoriatic arthritis, and acne).

SUPPLEMENTARY TABLE S2 Differentially expressed mRNAs between PRGs-mediated cluster 1 and cluster 2 of psoriasis samples.

SUPPLEMENTARY FIGURE S1 Differentially expressed mRNAs between psoriasis and normal samples. **(A)** Volcano plot of the training cohort of differentially expressed mRNAs. **(B)** Heatmap of the training cohort of differentially expressed mRNAs. **(C)** Volcano plot of the validation cohort of differentially expressed mRNAs. **(D)** Heatmap of the training cohort of differentially expressed mRNAs.

SUPPLEMENTARY FIGURE S2 The gene annotation analysis of the landscape of 39 PRGs using Metascape. **(A)**. The top-20 representative terms of gene annotation of PRGs. **(B)** A network based on subsets, colored by its terms ID. **(C)** A network based on subsets, colored by its *p*-values.

SUPPLEMENTARY FIGURE S3 The CIBERSORT **(A)** and immune cell abundance **(B)** of psoriasis compared with normal samples.
