# Supplementary figures and images for: Classification and biomarker gene selection of pyroptosis-related gene expression in psoriasis using a random forest algorithm
Source: Front Genet. 2022 Aug 30;13:850108. doi: 10.3389/fgene.2022.850108 (PMC9468882; doi:10.3389/fgene.2022.850108)

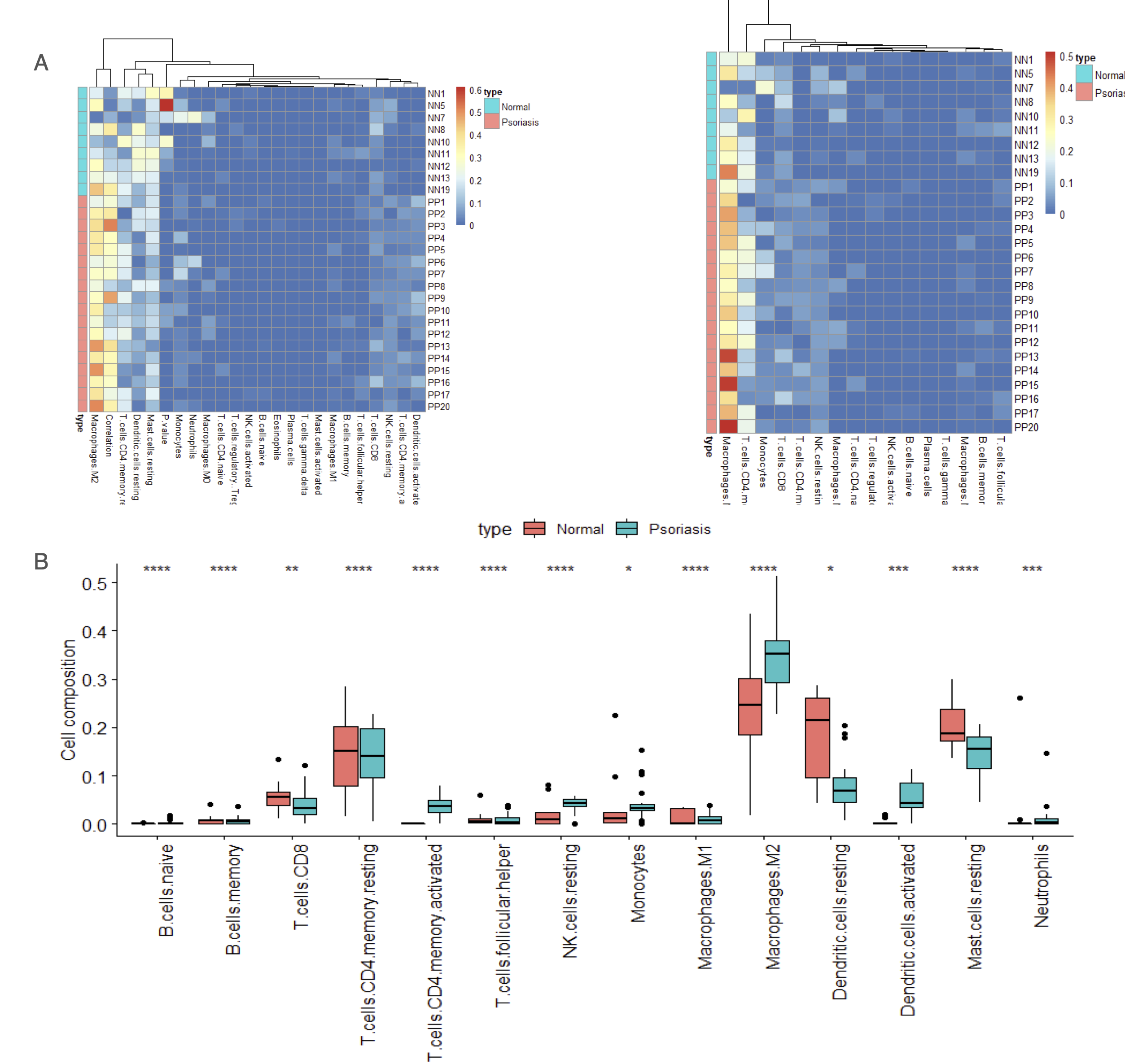

Supplement: Supplementary file 1 [file Image3.TIFF]

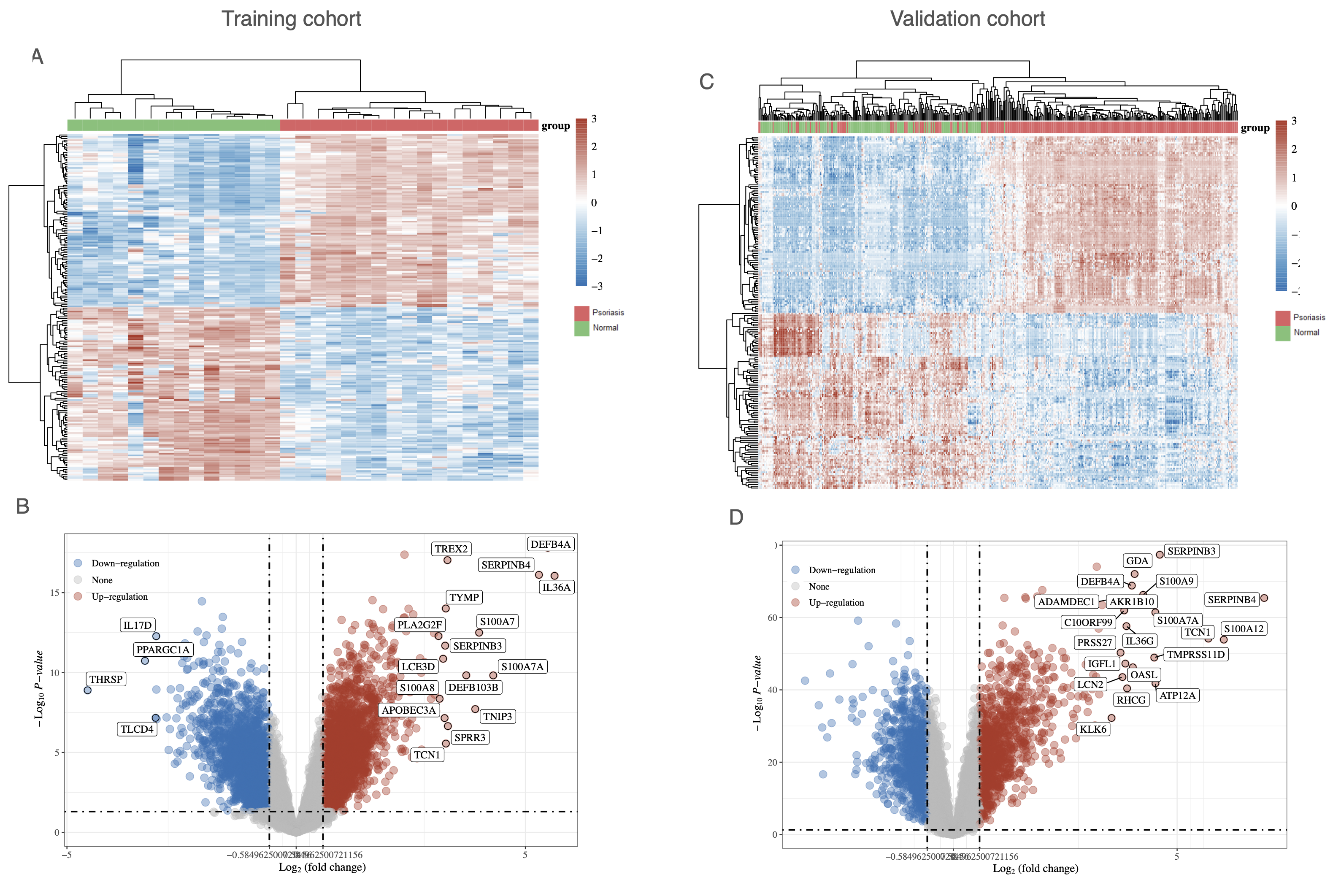

Supplement: Supplementary file 2 [file Image1.TIFF]

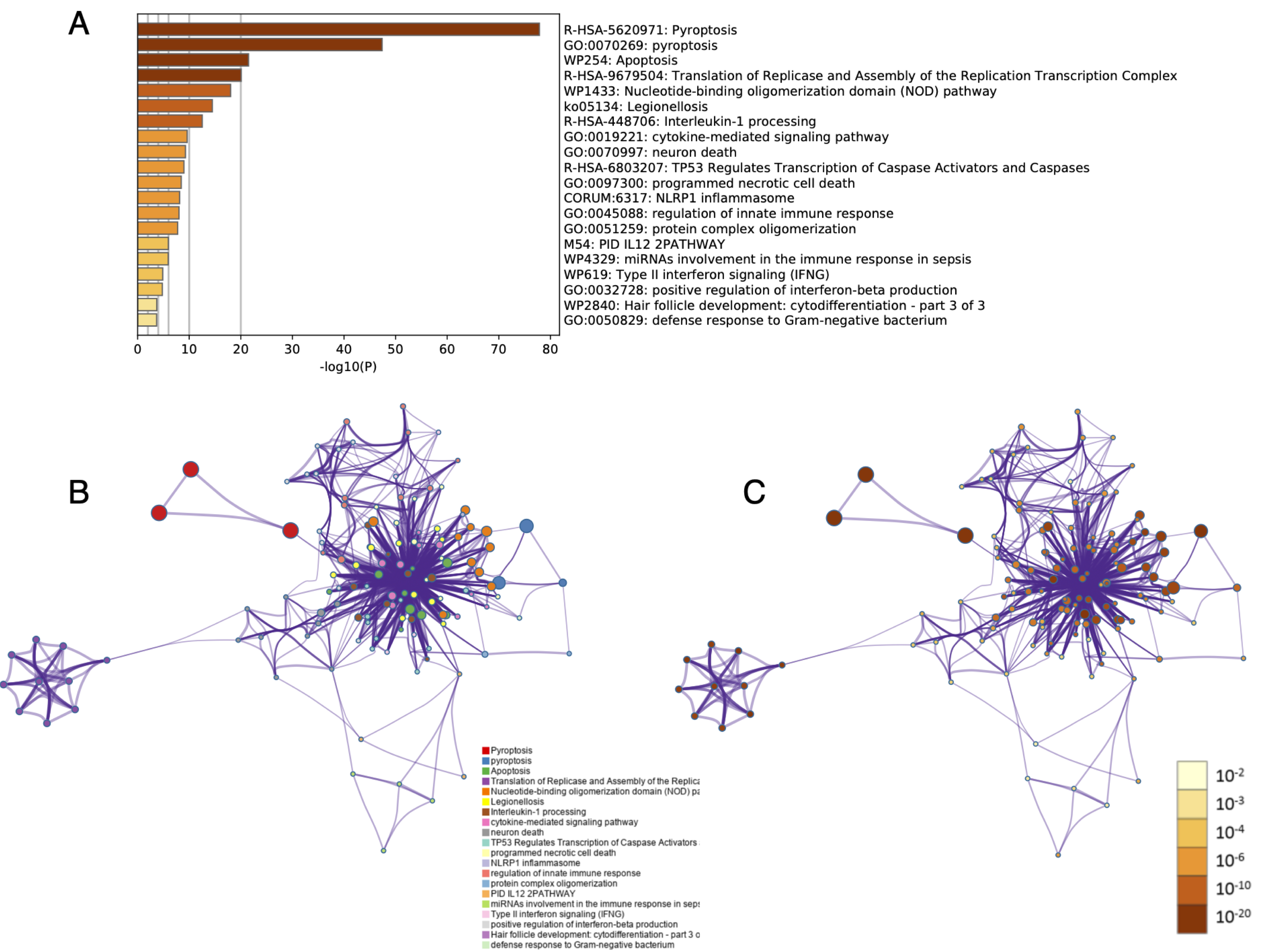

Supplement: Supplementary file 5 [file Image2.TIFF]
