# Supplementary material for: Classification and biomarker gene selection of pyroptosis-related gene expression in psoriasis using a random forest algorithm
Source: Front Genet. 2022 Aug 30;13:850108. doi: 10.3389/fgene.2022.850108 (PMC9468882; doi:10.3389/fgene.2022.850108)
Supplement: Supplementary file 4 [file DataSheet1.pdf]

上海中医药大学附属岳阳中西医结合医院
实验动物福利与伦理审查表

申请日期：2020 年 6 月 11 日　　　　伦理受理编号：YYLAC-2020-078-3

课题名称：基于系统生物学、网络药理学的“病-证”本质及中医药作用机制研究		
课题来源：国家科技部	课题编号：2018YFC1705305	课题级别：国家级
课题负责人：李斌	科室：皮肤科	
电话：18930568129	邮箱：18930568129 @163.com	
申请人：李斌	科室：皮肤病研究所	
电话：18930568129	邮箱：18930568129 @163.com	
实验参与人员：孙晓颖		

拟使用实验动物信息	动物来源：上海斯莱克实验动物有限责任公司	
	实验动物质量合格证编号：20170005028215	
	实验动物生产许可证编号：SCXK(沪)2017-0005	
	实验动物使用许可证编号：SYXK(沪)2018-0040	
	品种品系： C57	等级：SPF 级
	体重：	周龄：6-7w
	数量： 18 只（♂）	
	动物实验起止时间： 2020 年 06 月 16 日至 2020 年 06 月 30 日	

1、参与实验操作人员是否接触过实验动物并参加过相关培训？	[ ]否<br>[√]是（请提供具体说明）<br>(经岳阳医院动物中心纸质版培训)
2、该动物实验的安全性是否得到论证？	[ ]否<br>[√]是<br>负责人签字：[illegible]
3、该实验是否可选用更低等动物、离体器官、组织、细胞或其他无知觉材料替代哺乳动物？	[√]否<br>[ ]是（请提供具体原因）
4、该实验是否使用有毒（害）物质（感染、放射、化学毒、其他）？	[√]否<br>[ ]是（请提供具体原因）
5、该实验采取了何种减少动物用量、减轻动物痛苦、确保动物福利的措施？	[ ]否<br>[√]是（请提供具体原因）<br>①减少：施术者提升手术能力，提高动物实验利用率和实验精确度，尽可能减少实验所用动物数量。②优化：术区和饲养区分离以尽可能减少动物精神紧张和痛苦
6、实验设计及动物实验步骤	

请提供动物实验方案中各部分进一步的信息。包括实验目的、动物运输、动物饲养方式、实验操作步骤中等可能对动物产生伤害或不适的细节以及拟采取的防控措施。

实验目的：观察咪喹莫特银屑病模型小鼠银屑病样炎症反应的变化情况

饲养方式：正常饲养；☐单笼饲养；☐特殊饮食；☐特殊饮水；

☐其他（具体填写）

造模方法:

咪喹莫特软膏涂于小鼠双耳（共 62.5mg）进行造模 5 天。

给药方式：☐灌胃；☐腹腔注射；☐肌肉注射；☐静脉给药；☐无；

其他（具体填写）：咪喹莫特软膏外用

动物保定方式：徒手；☐固定板；

☐其他（具体填写）

麻醉（药物名称、剂量及给药途径）：

无。

主要观察指标：

组织病理，免疫组化及 PCR 等。

动物处死方法：

二氧化碳吸入室息法

声明：本人已认真审阅此申请表所填内容，保证所填内容真实可靠，并将严格遵守上述实验方案，自觉遵守实验动物福利伦理原则，随时接受委员会的监督与检查，按照申请原因正当使用审查批件，如违反规定，自愿承担一切后果。<br><br>声明人：<br>课题负责人签字：[illegible]　　动物实验负责人签字：[illegible]<br>2020年6月16日　　2020年6月16日
实验动物设施意见：<br>同意<br><br>设施主管签字：[illegible]　　设施负责人签字：[illegible]<br>2020年6月16日　　2020年6月16日
实验动物保护、福利、伦理委员会审批意见：<br>同意<br><br>主任签字（章）：[illegible]<br>2020年6月16日
